# Loneliness Predicts Development of Pain, Fatigue, and Depression in Older Adults

**DOI:** 10.1093/geroni/igab046.3233

**Published:** 2021-12-17

**Authors:** Victoria Powell, Navasuja Kumar, Mohammed Kabeto, Andrzej Galecki, Daniel Clauw, Maria Silveira

**Affiliations:** University of Michigan, Ann Arbor, Michigan, United States

## Abstract

Pain, fatigue, and depression form a well-recognized symptom cluster that is posited to have a shared mechanism. It is possible that chronic psychosocial stressors such as loneliness may impact the central nervous system and immune system, potentially leading to symptom cluster development. Loneliness is an increasingly recognized type of psychosocial stress, especially among older American adults. Thus, we investigated whether loneliness increased risk of developing the symptom cluster of pain, fatigue, and depression over time. Using Health and Retirement Study data from 2006 – 2016, we examined self-respondents ≥50 years-old for the presence of co-occurring pain, fatigue, and depressive symptoms surpassing threshold levels (ie., the symptom cluster). Loneliness (measured by the 3-item UCLA Loneliness Scale) at baseline was used as the predictor of interest. Of the total sample (n=11,766), n=5,956 (50.6%) had up to two complete sets of follow-up symptom cluster measurements. Logistic regression models for longitudinal data were fitted using a generalized estimating equation (GEE). After adjusting for demographic and clinical variables, loneliness strongly predicted the development of the symptom cluster (adjusted OR=3.16 (95% CI 2.77, 3.61)) as well as each component symptom (pain adjusted OR=1.54 (95% CI 1.42, 1.67); fatigue adjusted OR=1.88 (95% CI 1.74, 2.03); depression adjusted OR = 3.87 (95% CI 3.55, 4.21). Further research should investigate whether interventions targeting loneliness or other psychosocial stressors may have a role in prevention of this symptom cluster.

